# Assumptions and perceptions of food wasting behavior and intention to reduce food waste in the case of Generation Y and Generation X

**DOI:** 10.1038/s41598-025-86252-z

**Published:** 2025-01-23

**Authors:** László Mucha, Titanilla Oravecz

**Affiliations:** 1 Institute of Agricultural and Food Economics, Hungarian University of Agriculture and Life Sciences, 1. Páter K. Str., Gödöllő, 2100 Hungary; 2 Budapest Business University, 22-24. Diósy L. Str., Budapest, 1165 Hungary

**Keywords:** Food waste, Generation Y, Generation X, Theory of planned behavior

## Abstract

One of the global problems of our time is food waste that is most significant at the household level. There is a lack of research that focus on the food-wasting behavior of the main breadwinner groups in society, generations Y and X. To fill this gap, the purpose of this study is to analyse the factors that influence the food-wasting behavior of these groups. From data of a representative sample of 1,665 respondents by using structural equation modeling it is shown that the intention of reducing food waste is positively influenced by attitudes, health-, price and environmental consciousness, planning routines and ecological motives. The results show that influences from the immediate environment and the media are not effective, therefore programs dealing with the future of the Earth, children and sustainability, which strengthen environmental and ecological awareness and planning routines in generations X and Y are recommended.

## Introduction

Wasting food refers to the withdrawal of food from the food supply chain that is still fit for using, or expired or possibly damaged, caused by poor stock management economic behavior or ignore^1^. The value of food waste (FW) is estimated at $1 trillion per year globally, which represents a significant financial burden for the world’s inhabitants^2^. The EU imported 138 million tonnes of agrarian products worth €150 billion in 2021, while wasting around 153.5 million tonnes of food every year^3^. Aktas et al.^4^ highlight that FW is mainly characteristic of the later, consumer stages or retail of the supply chain. Research on FW has been given a new impetus by the COVID pandemic, which has been seen as a positive driver of FW avoidance behavior due to budget constraints and temporary food supply limitations^5^. Nowadays, it has become clear that the amount of FW is the most significant at the household level^6^, which necessitates a large number of empirical research examining consumer behavior^7^. Investigations into household wastage are particularly necessary in the EU, since 30% of the energy consumption of the EU countries is generated at the household level^8^. In order to prevent FW, it is crucial that professionals develop appropriate solutions taking the results of research into account^9^. Different generations have different consumption habits. The four main generations are Baby Boomers (1946–1964), Generation X (GX) (1965–1979), Generation Y (GY) or Millennials (1980–1994), and Generation Z (1995–2012)^10^. Karunasena et al.^11^ investigated the differences between the food-wasting behavior of the generations, focusing on young people’s attitudes towards FW. They found that FW was more prevalent among younger generations, caused by deficiencies in the areas of purchase, storage, and leftovers use. As Diniary et al.^12^ point out, empirical studies focusing on generational differences are still rare for understanding FW-related behavior. Further research on the structure of the food environment is needed to understand food waste^13^. Examining the food wastage behavior of Indonesian households, a different effect was observed in the case of Generation Y and Generation Z regarding guilt, religiosity and financial concern^12^. This research examines the food wasting behavior of Generations Y and X for the following reason. Generation X members prioritize spending on their household and family, they have a greater purchasing power than any other generation’s, and their consumer behavior is basically determined by caring for themselves and their family. Members of this generation hold important positions in both the entrepreneurial and public administration sectors. GY is diverse in every way, and that is what determines their consumer habits. Although their marriage rate is lower than that of GX, they are considered to be active earners and household breadwinners^14–16^. Purwanto et al.^17^ have found in the case of Generation X that attitude influences the intention to reduce FW, and directly and indirectly influences FW through the intention. Purwanto et al.^17^ propose as a future research direction to look over the impact of attitudes on the intention and behavior to reduce FW in GY. As a result of their systematic review analyzing empirical studies using the Theory of Planned Behavior (TPB) model, Etim et al.^18^ point out, it is essential to conduct researches as diverse and wide-ranging as possible for a global understanding of the nuances of FW. The study examining FW of Generation X^17^ did not make a generational comparison; moreover, it only examined the effect of attitude, without additional constructs. International literature lags behind in studies that focus on factors determining FW behavior (FWB) in GX and GY. The purpose of this study is to explore these factors.

To the best of our knowledge, there is no similar research as yet, therefore this study provides to the literature on household FW by using econometric modeling to investigate the effects of latent factors that influence the FWB and the intention to reduce food waste (IRFW) in the main breadwinner groups in society, GX and GY. The modeling includes factors whose impact on FW has not yet been studied. By identifying the impact of the large number of latent factors included in the research, this study makes recommendations to relevant professionals to help shape IRFW.

## Theoretical framework and hypotheses development

### Theory of Planned Behavior and hypotheses related to it

This study applies the TPB model, which has been used by several literature to investigate FW related behavior^19–21^, and their results suggest that TPB provides a powerful basis for describing food-wasting behavior^4,22,23^. According to a study using a systematic literature review and meta-analysis, the TPB is a particularly suitable framework for empirical studies dealing with FW^24^. TPB provides a framework to explain, understand and predict human behavior, the determinant of an individuals’ behavior is the intention whether to act or not and further affected by subjective norms (SN), attitude and perceived behavioral control (PBC) form the intentions^25^. TPB components like attitude, SN and PBC have a positive effect on the household’s IRFW^26,27^. The intention to avoid FW is determined significantly by attitudes towards FW^20,23,27–29^. The stronger the pressure of SN on individuals to reduce FW, the greater their willingness to participate^20^. SN positively influence IRFW, especially in relation to social media^30^. However, no significant effect of SN was observed when studying food wasting behavior of Indian university students^31^. PBC had a strong direct effect, and a weak indirect effect through intention on behavior^23^. PBC negatively influences IRFW and positively influences FWB^4,20^. PBC cannot determine intentions; it determines FWB through food-related routines^32^. The results are not consistent, the present study assumes that individual perceptions of the inevitability of FW negatively affect IRFW.

- H1/a: Attitude positively influences IRFW.
- H1/b: Attitude negatively influences FWB.
- H2/a: SN positively affect IRFW.
- H2/b: SN negatively affect FWB.
- H3/a: PBC negatively influences IRFW.
- H3/b: PBC positively influences FWB.
- H4: IRFW negatively influences FWB.

### Health consciousness

The health-conscious consumers value their health and they are willing to take action to protect it^33^, health consciousness (HC) is one of the drivers of healthy eating^34^. According to Barone et al.^28^ avoiding possible health risks and the goal of following healthy diet negatively influences IRFW. TPB was extended by HC in the study of Adel et al.^35^, according to their results, HC positively influences IRFW. Katt and Meixner^36^ found that HC had a direct and positive influence on FW prevention behavior.

H5/a: HC positively influences IRFW.

H5/b: HC negatively influences FWB.

### Environmental consciousness

Environmental consciousness (EC) positively affects the minimization of FW^37,38^, especially reducing FW^29^. The empirical results are not consistent, consumers who are environmentally conscious demonstrate positive waste prevention and recycling behavior, but not generate less FW^39^. EC has been often included as an extended element of TPB in FWB studies^20,26,40^. EC strongly affects consumer behavior related to FW minimization^41–44^, in contrast to others’ findings^32,45^. EC significantly influences the attitude toward FW minimization, which is connected with a higher level of behavior to reduce FW^46^. Szakos et al.^47^ found that environmentally conscious lifestyle was the most effective preventive factor in shaping FWB.

- H6/a: EC positively influences IRFW.
- H6/b: EC negatively influences FWB.

### Price consciousness

Consumer behavior on waste reduction is mostly influenced by saving money^48^. In general, consumers are sensitive to food prices^49^. Price-conscious consumers usually use shopping list, so price consciousness (PC) positively affect planning routines^50^. Extending the TPB by PC is widespread in FW research^4,28,35,36,46^. According to their results, PC has a significant effect in shaping IRFW. According to Pellegrini et al.^46^, PC positively affects the attitude that in turn affects to minimize FW. Katt and Meixner^36^ found that PC had a positive direct effect on FW prevention behavior.

- H7/a: PC positively influences IRFW.
- H7b: PC negatively affects FWB.

### Planning routines

Appropriate planning routines (PR) (e.g. checking food stocks at home, planning meals) ensure the reduction of FW^23^. Using a shopping list reduces FW^32^. According to Stancu et al.^23^, household PR affect FWB only indirectly, through shopping routines, in contrast to the research of Stefan et al.^32^. According to Ariyani and Ririh^20^, buying habits and household planning do not affect significantly the intention to manage FW; these findings are in contrast to the results of Visschers et al.^50^. PR reduce food surpluses, thus indirectly reduce FW^4^. Cammarelle et al.^51^ found that PR were important in understanding the consumer’s intention of reduction household FW.

- H8/a: PR positively influence IRFW.
- H8/b: PR negatively influence FWB.

### Ecological motives

Nowadays, concern for animal rights is receiving more and more attention, which, complemented by the environmental aspect can be well characterized by the concept of ecological motives (EM). Respecting animal rights and protecting the environment are important for consumers with EM^52^. The costumers with EM (ethical consumers) are more tended to buy ethical products^53^, they usually have strong involvement with organic foods^54,55^. The effect of this factor on food wastage has not yet been investigated, the present study fills this research gap. Since its impact has been primarily examined in relation to organic food, it is likely that organic thinking determines many other sustainability-related activities, including FW.

- H9/a: EM positively influence IRFW.
- H9/b: EM negatively influence FWB.

### Celebrations and holidays

The role of food in social relations has declined^56^. Changes in taste preferences within families lead to wastage^57^. National, family, friend celebrations and holidays (CH) affect the level of waste^56^. If eating routines change during a special period of the year (e.g. Ramadan) it leads to a higher level of FW^4^. Aktas et al.^4^ have identified other socio-cultural elements, such as the enhanced holidays like Christmas and Easter, as a future research direction for similar studies about FW. They also suggest including the examination of the effect of weddings or any family celebrations in empirical research on FW. This study continues this line of research.

- H10/a: CH negatively affect IRFW.
- H10/b: CH positively affect FWB.

### Unplanned events

The unplanned events (UE) may destroy the plans that households make to prevent FW^58^, therefore, due to lack of cooking and planning routines, UE can lead to unwanted household FW^59,60^. According to Farr‐Wharton et al.^61^, the reason for this is that unexpected events such as unexpected reasons for family members or unplanned gatherings and unexpected meals prevent planned meals. According to some research^57,61,62^, accelerated lifestyle leads to waste. Teng et al.^19^ investigated the moderating effect of UE by using TPB. According to their results, storage and cooking routines are not negatively related to FW, but at the same time, the effect of household storage and cooking routines is moderated by UE.

- H11/a: UE negatively affect IRFW.
- H11/b: UE positively affect FWB.

### Blaming others for food waste

The effort to reduce FW is of great importance at the household level, but consumers see the problem as global, so they feel less of their individual responsibility in helping to solve it, rather they expect it from others^63^. Kim et al.^40^ investigated the IRFW of restaurant customers, according to their results, the moral norm for FW reduction, the responsibility for FW and awareness of environmental impacted as predictors for IRFW. To explore the causes of FW, Pocol et al.^64^ investigated the respondents’ awareness of the issue. They found that since FW was highest at the household level, not only it is important to examine whether consumers are aware of this or not, but also to clarify the impact of individual responsibility shifting on actual FW. According to Graham-Rowe et al.^63^, preventing FW lacks a sense of individual responsibility. The causal effect of the blaming others for food waste (BO) factor has not yet been investigated in relation to FW, this study fills this research gap.

H12/a: BO negatively influences IRFW.

H12/b: BO positively influences FWB.

### Generations Y and X

The amount of FW is clearly determined by household size, households with multiple members produce a larger amount of FW overall, while households with one person lead a more wasteful lifestyle^65,66^. Due to different consumption habits and attitudes towards FW, young people waste food more often and in greater quantities than older people^32,66,67^. Young people often do not yet understand the value of food, which is why they throw away more food^67^. Younger average age households produce more FW and do not use shopping lists when shopping^29^. FW among members of younger generations is caused by deficiencies experienced in the areas of purchase, storage and residual use^11^, authors relate this fact to PR. For 18–24 year olds, the lack of making shopping lists and planning weekly meals is also responsible for higher FW^11,68^. Furthermore, it is difficult for young consumers not to buy more than necessary and not to prepare too much food due to the guests during various holidays^69^. It is important to highlight that these findings apply to consumers younger than GY, but according to this study, they are also likely to apply to Millennials. GX members have been characterized by self-care since childhood, which is complemented by the motivation to take care of the family as adults, and their financial awareness fundamentally shapes their attitudes^14–16^.

- H13/a: Generations moderate the link between PR and IRFW, such as the relationship is stronger in the case of X generation.
- H13/b: Generations moderate the link between PC and attitude to reduce FW, such as the relationship is stronger in the case of X generation.

**Fig. 1:**
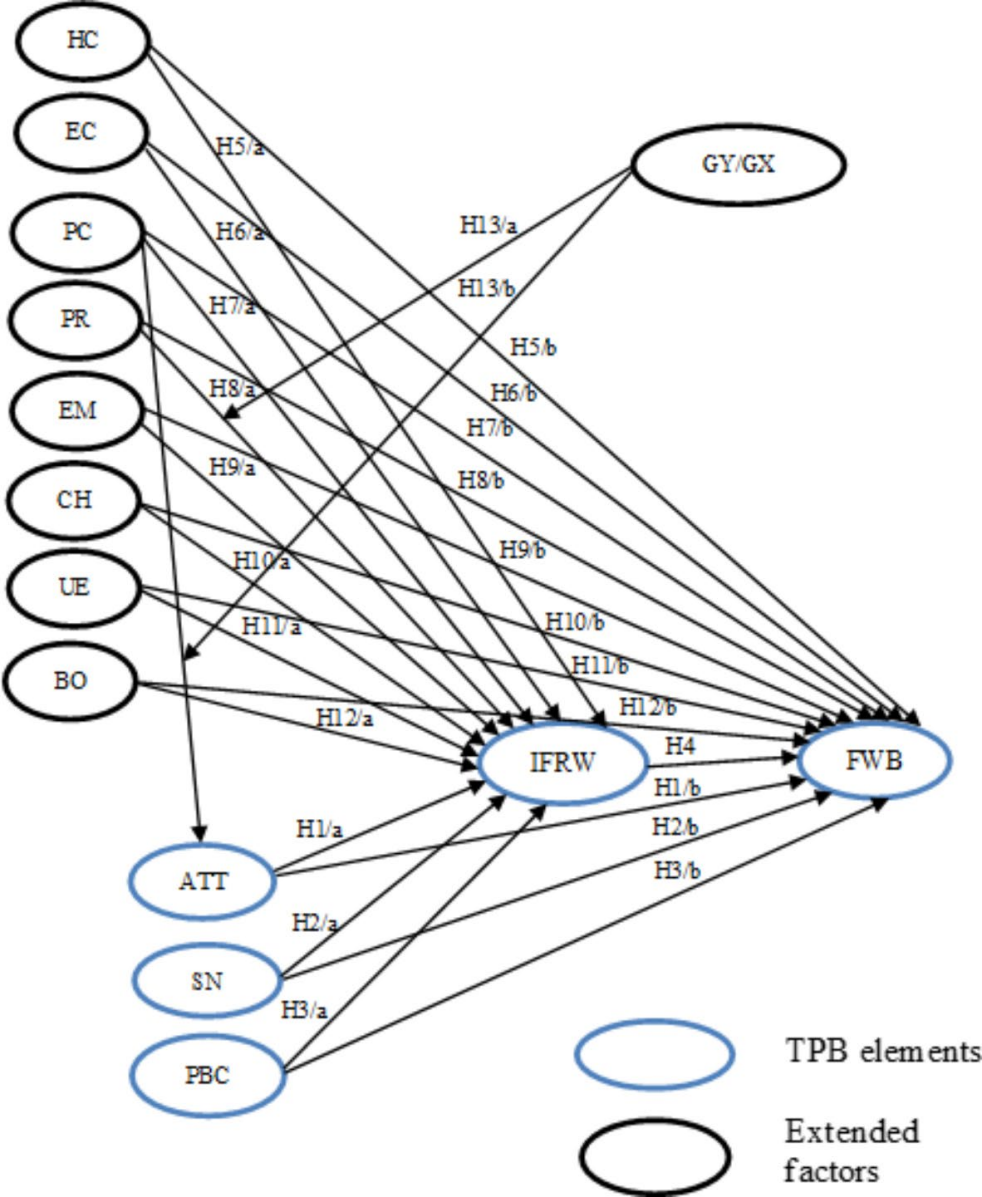
Study framework with hypotheses. ATT: attitude SN: subjective norms, PBC: perceived behavioral control, IRFW: intention to reduce food waste, FWB: behavior of food waste, HC: health consciousness, EC: environmental consciousness, PC: price consciousness, PR: planning routines, EM: ecological motives, CH: celebrations and holidays, UE: unplanned events, BO: blaming others for food waste, GY/GX: Generation Y / Generation X.

The study framework with hypotheses is shown in Fig. 1.

## Data and methodology

### Procedure and sample

Data were collected by face-to-face interviews applying the LimeSurvey platform from 4th April to 6th May in 2022. The survey collected information from Hungarian consumers of GY and GX, who are responsible for both their own and their household’s food purchases, as well as their household’s food consumption and waste. A quota sampling method was used to collect data to provide an appropriate estimate of the populace features^70^. Quota variables were gender and generation. Interlocking quotas was defined^71^ for a sample of 1700 people. The rate of the quotas was continuously compared to the general population; the final sample contained 1665 individuals. Interlocking quotas met, the sample is representative in terms of gender and GY and GX of the adult population of Hungary (Table 1).

**Table 1:** Proportion and representativeness of the sample.

		Respondents*		Hungarian population**	
		No	%	No	%
Gender	Female	831	49.91	2,043,011	49.27
	Male	834	50.09	2,103,457	50.73
Age	Y Generation (26–40 years)	878	52.73	1,933,635	53.37
	X Generation (41–55 years)	787	47.27	2,212,833	46.63

Representativeness of the sample: gender and generation Y and X (χ2 = 0.759, df = 3, *p* = 0.859), *n = 1,665, **^72,73^.

### Ethical statement

All methods were carried out in accordance with relevant guidelines and regulations; Ethical approval was obtained from the Ethical Committee of the Budapest Business University. The participation of the respondents was entirely consensual and anonymous, with informed consent; all participants accepted and voluntarily participated in the study.

### Questionnaire design and measures

The questionnaire was designed based on scales validated in previous research (Table 2). To find out about FWB, respondents were questioned to rate on a scale of 1 to 5 how often they throw out food (1: never; 2: rarely; 3: average; 4: often; 5: very often) for five types of the most frequently thrown away food in Hungary^47,74^. As several researches point out^30,75,76^, nowadays social media celebrities are important actors in the media space, as social media has provided a platform for people to connect on a global scale. Therefore, a specific question in the questionnaire is dedicated to investigate the influence of famous people in social media. To measure latent factors the respondents expressed their acceptance with the measurement items on a five-point scale (strongly disagree, disagree, neither disagree or agree, agree and strongly agree). Two English-Hungarian native speakers translated the scale items, developed in English, into Hungarian. They confirmed the consistency between the translated content and the original content. To avoid common method bias (CMB), the questions were shown to the respondents in a shuffled manner, and they were informed that there are no right or wrong answers^77^.

**Table 2:** Constructs, measurement items and sources.

Constructs	Measurement item		Sources
Food Waste Behavior (FWB)	FWB1	Meals and leftovers	Stancu et al.^23^, Kasza et al.^74^, Szakos et al.^47^
	FWB2	Bread and other bakery products	
	FWB3	Dairy products	
	FWB4	Fresh fruits and vegetables	
	FWB5	Processed meat	
Intentions (INT)	INT1	I intend to eat leftover food.	Aktas et al.^4^, Visschers et al.^50^, Stefan et al.^32^, Stancu et al.^23^
	INT2	I intend not to throw away food.	
	INT3	I intend to generate as little food waste as possible.	
	INT4	I intend to find a use for food trimmings.	
Attitude (ATT)	ATT1	I feel bad when uneaten food is thrown away.	Aktas et al.^4^, Stancu et al.^23^, Ajzen^78^
	ATT2	I was raised to believe that food should not be wasted.	
	ATT3	Throwing away food bother me.	
	ATT4	In my opinion wasting food is extremely negative.	
	ATT5	In my opinion loading the environment with my household’s food waste is extremely negative.	
Subjective Norms (SN)	SN1	My friends and my family influence my opinion about and behavior in reducing food waste.	Aktas et al.^4^, Ajzen^78^
	SN2	Influenced by the media (TV, radio, Facebook, Instagram, Youtube) or well-known people (influencers), I pay attention to throwing away as little food as possible.	
	SN3	I believe that media (TV, radio, Facebook, Instagram, Youtube) or well-known people influence my opinion and behavior regarding food waste.	
	SN4	Society thinks my efforts to reduce food waste are necessary.	
Perceived	PBC1	In my opinion wasting food is unavoidable.	Teng et al.^19^, Stancu et al.^23^
Behavioral	PBC2	In my opinion loading the environment with my household’s food waste is unavoidable.	
Control (PBC)	PBC3	Not to throw food away would be difficult.	
Health Consciousness (HC)	HC1	I carefully choose food to ensure good health.	Michaelidou and Hassan^79^, Adel et al.^35^, Teng and Lu^80^, Katt and Meixner^36^)
	HC2	I’m usually aware of my health.	
	HC3	I’m very self-conscious about my health.	
	HC4	I think often about health issues.	
	HC5	I take responsibility for the state of my health.	
Environmental Consciousness (EC)	EC1	The current development path is destroying the environment.	Katt and Meixner^36^
	EC2	Unless we do something, environmental damage will be irreversible.	
	EC3	Food consumption level can have an impact on the environment.	
Price Consciousness (PC)	PC1	I try to buy food items that are on sale.	Katt and Meixner^36^, Pellegrini et al.^46^
	PC2	I pay attention to good deals.	
	PC3	I compare food prices from different brands.	
	PC4	Spending time to find the most affordable price.	
	PC5	Despite I prefer those brands I always buy discounted brands.	
Planning Routines (PR)	PR1	I check my cupboard/fridge/pantry prior to a trip to the supermarket.	Aktas et al.^4^, Stancu et al.^23^, Özbük et al.^49^,
	PR2	I plan my meals in advance and keep to my plan.	
	PR3	I plan what to eat to ensure I use the most short-dated food first.	
Ecological Motives (EM)	EM1	It’s very important that the foods have been produced in a way that animals’ rights have been respected. (e.g., sufficient living spaces).	Teng and Lu^80^
	EM2	It’s very important that the foods have been prepared in an environmentally friendly way.	
	EM3	It’s very important that the foods are packaged in an environmentally friendly way.	
	EM4	It’s very important that the foods have been produced in a way which has not shaken the balance of nature.	
Celebrations and Holidays (CH)	CH1	I feel that I throw away food more than usual during Holidays.	Aktas et al.^4^
	CH2	During Holidays, the food I prepare for guests is wasted.	
	CH3	My food preferences during Holidays result in higher waste than other times of the year.	
Unplanned Events (UE)	UE1	Due to unexpected dining out, I always have to change my family cooking plan.	Farr‐Wharton et al.^61^, Teng et al.^19^
	UE2	Due to unexpected parties, I always have to change my family cooking plan.	
	UE3	Due to unexpected reasons from my family members, I always have to change my family cooking plan (e.g., someone cannot dine at home without prior notice).	
Blaming others for food waste (BO)	BO1	Who do you think is responsible for food waste? [Government institutions]	Pocol et al.^81^
	BO2	Who do you think is responsible for food waste? [School canteens]	
	BO3	Who do you think is responsible for food waste? [Restaurants]	
	BO4	Who do you think is responsible for food waste? [Hypermarkets]	

### Methodology

Structural equation modeling (SEM) method was used in the study, which was carried out in steps in accordance with the literature. The validity of convergence and reliability were checked by using the average variance extracted index (criterion AVE > 0.5) and the composite reliability index (criterion CR > 0.7). The reliability of the scales was tested by using Cronbach’s alpha (criterion α > 0.7), maximum shared variance (MSV) was also checked by using the AVE > MSV criteria^82,83^. Discriminant validity was checked in two ways. Firstly, by comparing the correlation between factors and the square root of the AVE^84^, where the square root of the AVE must exceed the correlations. Secondly, by using a Heterotrait-monotrait ratio of correlations (HTMT) analysis, where the correlation must not exceed 0.85^85^. Absolute and comparative indices were used to determine the appropriate model fit. Cut-off values were defined as: *p* > 0.05; CMIN/DF < 3; RMSEA < 0.06, SRMR < 0.08; GFI, CFI, NFI, TLI > 0.90^82,86^. To examine the moderating effect of generations, Multi Group Analysis (MGA) was used, when unconstrained model is compared with the measurement weights model. The moderating effect of the investigated variables is confirmed in the case of the significant Chi-square test^87^. Excel, IBM SPSS Statistics 22.0 and IBM AMOS 24 software were used to analyse data. Maximum Likelihood estimation was used for SEM, and indirect effects were tested by using a Bootstrap procedure (5000; CI: 95%).

## Results

### Testing the measurement model and common method bias test

Table 3 shows the reliability, convergent validity and the Fornell-Larcker criterion for discriminant validity in the case of the measurement model. For FWB, the AVE is 0.414, for BO, AVE is 0.480 which, considering the CR values (0.779 and 0.781), according to^84^ and^70^, are still acceptable. For the other variables the criteria defined in the Methodology chapter were fulfilled. HTMT criteria, was also fulfilled (Table 4). The model had good fit (Table 3).

**Table 3:** Reliability, convergent validity and Fornell-Larcker criterion for discriminant validity.

		CR	AVE	MSV	α	1	2	3	4	5	6	7	8	9	10	11	12	13
1	FWB	0.779	0.414	0.215	0.778	**0.643**												
2	IRFW	0.826	0.545	0.512	0.809	-0.295***	**0.738**											
3	SN	0.819	0.545	0.130	0.809	0.087**	0.101***	**0.738**										
4	ATT	0.848	0.532	0.512	0.856	-0.180***	0.716***	0.155***	**0.729**									
5	PBC	0.802	0.576	0.207	0.800	0.455***	-0.277***	0.089**	-0.266***	**0.759**								
6	EM	0.875	0.638	0.327	0.869	-0.134***	0.449***	0.123***	0.572***	-0.240***	**0.799**							
7	HC	0.850	0.536	0.198	0.851	-0.191***	0.296***	0.245***	0.312***	-0.168***	0.374***	**0.732**						
8	PC	0.854	0.541	0.103	0.866	-0.005	0.249***	0.280***	0.241***	0.017	0.099***	0.179***	**0.735**					
9	BO	0.781	0.480	0.088	0.730	0.081**	0.194***	0.106***	0.242***	-0.034	0.243***	0.124***	0.099***	**0.693**				
10	EC	0.822	0.609	0.318	0.816	-0.125***	0.435***	0.065*	0.564***	-0.217***	0.561***	0.201***	0.171***	0.297***	**0.780**			
11	CH	0.844	0.645	0.215	0.836	0.464***	-0.139***	0.168***	-0.111***	0.324***	-0.128***	-0.010	0.102***	-0.006	-0.111***	**0.803**		
12	UE	0.914	0.781	0.152	0.914	0.279***	-0.080**	0.360***	-0.021	0.195***	-0.010	0.086**	0.191***	-0.004	-0.077**	0.390***	**0.883**	
13	PR	0.770	0.532	0.198	0.765	-0.180***	0.402***	0.261***	0.384***	-0.165***	0.248***	0.445***	0.321***	0.124***	0.216***	-0.014	0.146***	**0.730**

Data in bold show square root of AVE. CR: composite reliability index, AVE: average variance extracted, MSV: maximum shared variance, α: Cronbach’s alpha, ATT: attitude SN: subjective norms, PBC: perceived behavioral control, IRFW: intention to reduce food waste, FWB: behavior of food waste, HC: health consciousness, EC: environmental consciousness, PC: price consciousness, PR: planning routines, EM: ecological motives, CH: celebrations and holidays, UE: unplanned events, BO: blaming others for food waste. ***p < 0.001, **p < 0.05. Model fit: χ2 = 3200.865, df = 1138, *p* < 0.001, CMIN/DF = 2.813, GFI = 0.926, TLI = 0.942, CFI = 0.948, NFI = 0.922, RMSEA = 0.033, PClose = 1.000, SRMR = 0.044.

**Table 4:** Heterotrait-Monotrait Ratio of Correlations (HTMT) criterion for discriminant validity.

		1	2	3	4	5	6	7	8	9	10	11	12	13
1	FWB													
2	IRFW	0.311												
3	SN	0.092	0.181											
4	ATT	0.159	0.696	0.259										
5	PBC	0.463	0.296	0.110	0.259									
6	EM	0.140	0.438	0.169	0.574	0.245								
7	HC	0.185	0.317	0.277	0.347	0.170	0.400							
8	PC	0.021	0.280	0.282	0.278	0.000	0.123	0.199						
9	BO	0.089	0.246	0.142	0.313	0.046	0.298	0.163	0.141					
10	EC	0.122	0.439	0.119	0.576	0.219	0.579	0.240	0.204	0.393				
11	CH	0.471	0.153	0.187	0.099	0.343	0.120	0.012	0.094	0.003	0.116			
12	UE	0.287	0.074	0.365	0.002	0.200	0.004	0.090	0.171	0.007	0.074	0.418		
13	PR	0.180	0.454	0.276	0.416	0.167	0.275	0.438	0.329	0.160	0.264	0.021	0.136	

ATT: attitude SN: subjective norms, PBC: perceived behavioral control, IRFW: intention to reduce food waste, FWB: behavior of food waste, HC: health consciousness, EC: environmental consciousness, PC: price consciousness, PR: planning routines, EM: ecological motives, CH: celebrations and holidays, UE: unplanned events, BO: blaming others for food waste.

CMB was checked in three steps. Firstly, the Harman single factor test was used; the total variance explained by the variables was 17.78%, which is less than the recommended threshold value of 50%. Secondly, a single factor was run using confirmatory factor analysis (CFA), the model showed a poor fit (χ^2^ = 21.105, df = 1216, *p* < 0.001, CMIN/DF = 21.105, GFI = 0.531, TLI = 0.351, CFI = 0.383, NFI = 0.373, RMSEA = 0.110, PClose = 0.001, SRMR = 0.124), indicating a lack of CMB^88^. In the third step, a common method factor was included in the model, and all the relationships in the common method construct model were constrained to be equal. The difference between the CFA performed on the two models (with and without the common method factor) was 1 degree of freedom (df). The difference in chi-squared values from the CFA of the two models exceeded the threshold of 3.84 (significance of 1 df is 3.84 at the *p* = 0.05 level), indicating the presence of a CMB. Therefore, the factor scores for each model factor (including the common method variable) were saved by data imputation, which allowed controlling the bias of the common method during the path analysis^89^.

### Testing the structural model and hypotheses H1-H13

The structural model was constructed from the factor scores generated by imputing the data, taking into account the common method variable, based on the conceptual model. Modification Indices were also taken into account. The model included FWB, IRFW, attitude and PBC as endogenous variables. The model had excellent fit (Table 5). Table 5 shows the direct, indirect and total effects in the model, results of hypotheses testing. The indirect effects are significant, if the confidence interval does not include zero. Due to the large number of model factors, a number of indirect effects were also generated; Table 6 shows the other standardized direct effects in the model associated with the hypotheses.

**Table 5:** Direct, indirect and total effects in the model, results of hypotheses testing.

Hypothesized relationship				Standardized direct effects, *p* value	Standardized indirect effects, standard error (SE), confidence interval (CI)	Standardized total effects, * p* value	Confirmation of expectation
H1/a	ATT	→	IRFW	0.468***	0.131 (SE = 0.011; CI = 0.110–0.153)	0.599***	Supported
H1/b		→	FWB	0.226***	-0.394 (SE = 0.022; CI = -0.438–0.350)	-0.168***	Supported
H2/a	SN	→	IRFW		-0.032 (SE = 0.009; CI = -0.050–0.014)	-0.032***	Not
H2/b		→	FWB	0.017***	-0.017 (SE = 0.005; CI = -0.028–0.007)		Not
H3/a	PBC	→	IRFW	-0.539***		-0.539***	Supported
H3/b		→	FWB	-0.824***	0.535 (SE = 0.029; CI = 0.479–0.590)	-0.289***	Not
H4	IRFW	→	FWB	-0.992***		-0.992***	Supported
H5/a	HC	→	IRFW	-0.170***	0.139 (SE = 0.011; CI = 0.119–0.164 )	-0.030 n.s	Not
H5/b		→	FWB	-0.556***	0.243 (SE = 0.019; CI = 0.205–0.280)	-0.313***	Supported
H6/a	EC	→	IRFW	-0.215***	0.314 (SE = 0.020; CI = 0.274–0.355)	0.098***	Supported
H6/b		→	FWB	-0.565***	0.263 (SE = 0.021; CI = 0.221–0.303)	-0.302***	Supported
H7/a	PC	→	IRFW		0.107. (SE = 0.017; CI = 0.075–0.142)	0.107***	Supported
H7/b		→	FWB	-0.177***	0.009 (SE = 0.007; CI = -0.004–0.142)	-0.168***	Supported
H8/a	PR	→	IRFW	0.069***	0.225 (SE = 0.017; CI = 0.193–0.260)	0.295***	Supported
H8/b		→	FWB	-0.232***	-0.034 (SE = 0.018; CI = -0.069–0.003)	-0.266***	Supported
H9/a	EM	→	IRFW	-0.119***	0.294 (SE = 0.019; CI = 0.258–0.333)	0.175***	Supported
H9/b		→	FWB	-0.290***	0.133 (SE = 0.019; CI = 0.096–0.171)	-0.157***	Supported
H10/a	CH	→	IRFW	-0.178***	-0.029 (SE = 0.017; CI = -0.062–0.006)	-0.207***	Supported
H10/b		→	FWB	-0.036***	0.214 (SE = 0.019; CI = 0.178.-0.251)	0.178***	Supported
H11/a	UE	→	IRFW	-0.196***	0.049 (SE = 0.011; CI = 0.028–0.070)	-0.147***	Supported
H11/b		→	FWB	-0.213***	0.221 (SE = 0.018; CI = 0.187–0.258)	0.008 n.s.	Not
H12/a	BO	→	IRFW	-0.181***	0.126 (SE = 0.010; CI = 0.106–0.147)	-0.055***	Supported
H12/b		→	FWB	-0.354***	0.247 (SE = 0.018; CI = 0.212–0.282)	-0.107***	Not

ATT: attitude SN: subjective norms, PBC: perceived behavioral control, IRFW: intention to reduce food waste, FWB: behavior of food waste, HC: health consciousness, EC: environmental consciousness, PC: price consciousness, PR: planning routines, EM: ecological motives, CH: celebrations and holidays, UE: unplanned events, BO: blaming others for food waste. ***p < 0.001, n.s.: not significant. Model fit: χ2 = 15.018, df = 6, *p* = 0.020, CMIN/DF = 2.503, GFI = 0.999, TLI = 0.993, CFI = 0.999, NFI = 0.999, RMSEA = 0.030, PClose = 0.955, SRMR = 0.009. Standardized estimates, confidence intervals, *p* values, model fit indices are the results of SEM AMOS processing.

**Table 6:** Standardized direct effects in the model associated with the hypotheses.

Relationship			Standardized direct effects, * p* value	Relationship			Standardized direct effects, * p* value
ATT	→	PBC	-0.243***	PR	→	ATT	0.176***
BO	→		-0.234***	EC	→		0.243***
EC	→		-0.312***	EM	→		0.291***
HC	→		-0.258***	PC	→		0.099***
PR	→		-0.223***	CH	→		-0.107***
PC	→		-0.089***				
UE	→		-0.092***				
EM	→		-0.222***				
SN	→		0.059***				
CH	→		-0.065***				

ATT: attitude SN: subjective norms, PBC: perceived behavioral control, HC: health consciousness, EC: environmental consciousness, PC: price consciousness, PR: planning routines, EM: ecological motives, CH: celebrations and holidays, UE: unplanned events, BO: blaming others for food waste. ***p < 0.001.

Attitude plays a very important role in influencing IRFW and FWB. Attitudes are positively influenced by respondents’ EC, PC, PR and EM, while CH weaken positive attitudes towards FW (Table 6). The inevitability of FW (PBC) strongly weakens IRFW, but the positive effect on behavior is not confirmed by the results. PBC is shaped by a number of model elements (Table 6), which shows that the inevitability of FW, as a subjective perception, is determined by the individual’s attitude and mindset. IRFW is a very strong predictor of reducing FWB. Looking at the effect of a large number of exogenous variables, it can be concluded that the respondents’ HC, EC, PC, PR, EM and attitude are the most important factors in positively shaping IRFW and in reducing FWB. The model did not confirm the role of environmental influence (SN). CH, as well as UE, have a negative effect on intention, however, out of the two factors, only celebration events and holiday were clearly responsible for FWB. BO does not increase FWB either, but it has a negative effect on IRFW.

**Fig. 2:**
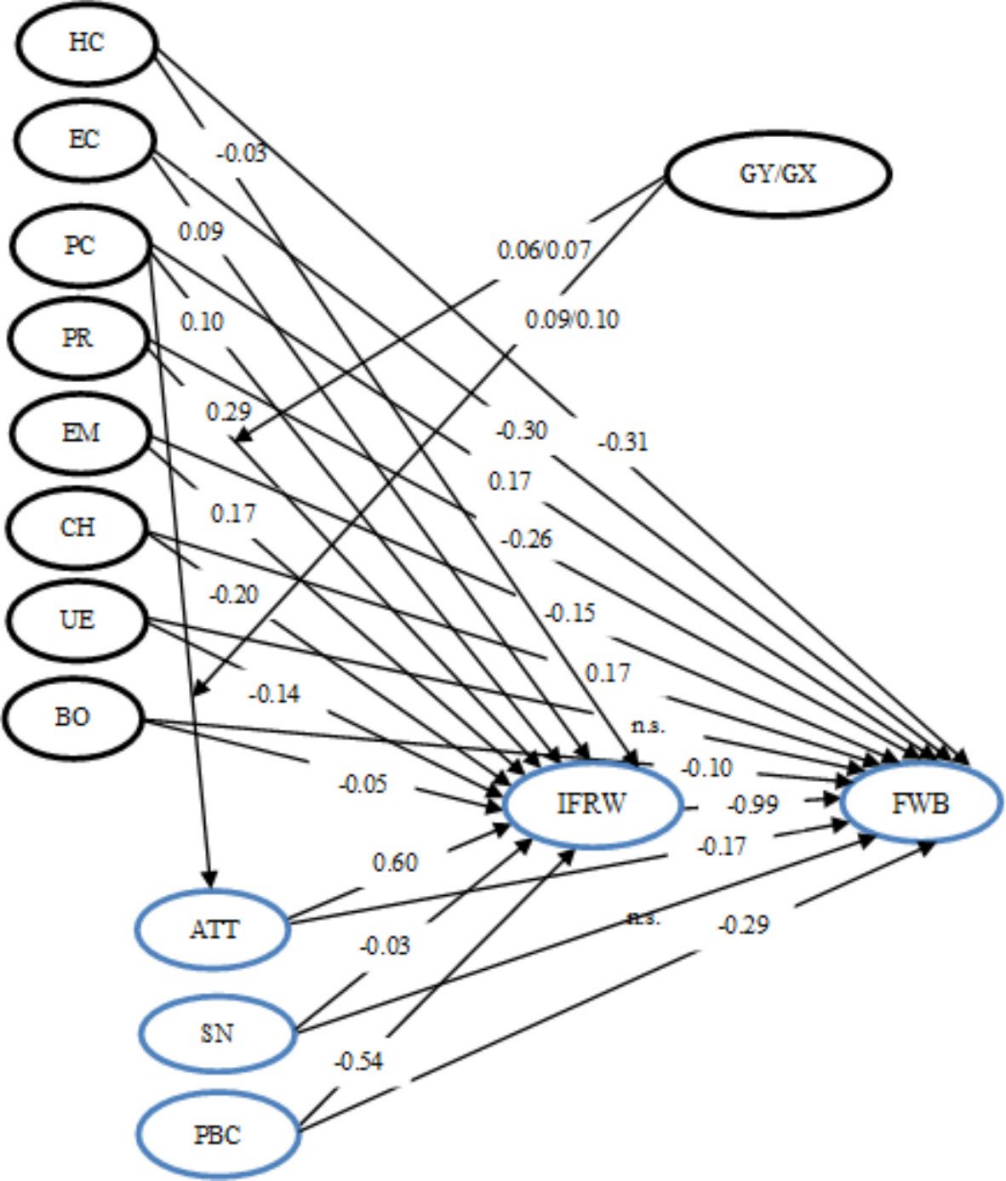
Results of hypotheses testing. Arrows indicate total effects with the value of the standardized total effect coefficients. ATT: attitude SN: subjective norms, PBC: perceived behavioral control, IRFW: intention to reduce food waste, FWB: behavior of food waste, HC: health consciousness, EC: environmental consciousness, PC: price consciousness, PR: planning routines, EM: ecological motives, CH: celebrations and holidays, UE: unplanned events, BO: blaming others for food waste, GY/GX: Generation Y/ Generation X, n.s.: not significant.

The differences in FWB between GY and GX were examined by using the Mann–Whitney (MW) test. There is no significant difference between the two generations only in the frequency of throwing away fresh fruit and vegetables out of the five food groups examined in the study. Millennials waste significantly more than GX members in the case of meals and leftovers Y(Mdn = 3), X(Mdn = 3); MW: U = 305,790.000, Z = -4.211, *p* < 0.001 (1-tailed), r = 0.103); bread and other bakery products (Y(Mdn = 3), X(Mdn = 2); MW: U = 327,252.500, Z = -1.932, *p* = 0.026 (1-tailed), r = 0.047); dairy products (Y(Mdn = 2), X(Mdn = 2); MW: U = 304,137.500, Z = -4.451, *p* < 0.001 (1-tailed); r = 0.110); processed meat (Y(Mdn = 2), X(Mdn = 1); MW: U = 324,596.000, Z = -2.243, *p* = 0.012 (1-tailed), r = 0.055). The first basic condition of MGA is the configural invariance test, which expresses that the factor weight matrices are identical for all groups^89^. Strong model fits across both groups were present (χ^2^ = 4516.768; df = 2276; *p* < 0.001; CMIN/DF = 1.985; GFI = 0.901; TLI = 0.937; CFI = 0.944; NFI = 0.893; RMSEA = 0.024), which shows that the data is invariant across the groups from a configural or structural perspective. In the second step, by metric invariance was tested whether the indicators measure the same construct in the same way in different groups, the analysis hypothesizes that the measurement weights model of the groups has a significant difference. The result (χ^2^ = 31.617; df = 38; *p* = 0.758) shows that there are no differences in how to conceptualize one or more of the theoretical concepts composing the model between Y and X generations. To analyse the categorical moderating effect of the generations, one structural relationship at a time was constrained to measure if the specific relationship is different across the groups. Model had excellent fit across the groups: χ^2^ = 23.938, df = 12, *p* = 0.021, CMIN/DF = 1.995, GFI = 0.998, TLI = 0.990, CFI = 0.999, NFI = 0.998, RMSEA = 0.024. Although in the case of Generation X the standardized direct effect of planning routines on intention is higher (β = 0.072***) than in the case of GY (β = 0.063**), there is no significant difference between the effects (∆ χ2/1df = 0.124, *p* = 0.725), which confutes H13/a. Although in the case of Generation X the standardized direct effect of price consciousness on attitude is higher (β = 0.108***) than in the case of GY (β = 0.091***), there is no significant difference between the effects (∆ χ^2^/1df = 0.218, *p* = 0.640), which confutes H13/b. The model explains 67.6% of the variance of IRFW and 99.6% of the variance of FWB. For Generation X, the rates are 68.6% and 99.7% respectively, while for Millennials the rates are 66.4% and 99.6%. The results of hypotheses testing are shown in Fig. 2.

## Discussion

### Theoretical contributions

The recycling and disposal of food has accompanied human history, but the increasing scale of FW requires an understanding of the phenomenon and the underlying structures^13,90^. Although FW has been a topic of international forums’ agenda since 1979, it only came to the focus of the mainstream interest after 2011^91,92^. FW has been steadily increasing in recent years, with total food waste in households, retail and food services increasing by 120 million tonnes from 2019 to 2022, and at the household level increasing from 121 kg per capita in 2019 to 132 kg per capita in 2024^93^. As Diniary et al.^12^ point out, empirical studies focusing on generational differences are still rare for understanding FW-related behavior. This is the first study that examines FWB of generations X and Y by including a large number of latent factors in econometric modeling to explain IRFW and FWB. Thus, it contributes to the literature on household FW and complements it by helping to understand FWB of the main breadwinner groups in society, generations X and Y, and identifying factors that increase the intention to avoid it. In the extended TPB model, the explained variance is very high for both intention (67.6%) and behavior (99.6%), compared to similar previous studies.

Ghani et al.^94^ were able to explain only 13.7 percent of the variance in IRFW, they concluded that other components not included in the study influence intention. Graham-Rowe et al.^95^ explained only 8 percent of the variance in the intention of reducing household vegetable and fruit waste. The result of Chalak et al.^96^ explained 68.4 per cent of FWB, the combined FW model of Stancu et al.^23^ explained 45 per cent for intention and 43 per cent for FWB. Findings of Russell et al.^97^ explained 46% of the variance in FWB, while the extended TPB model of Aktas et al.^4^ explained 35%. Similar results were obtained by Barone et al.^28^ (56% explained variance). When examining IRFW, Attiq et al.^98^ found a similar result to the present study (65%). The results of the present study support previous research^4,22,23^ that considers TPB as an appropriate conceptual structure for the study of FWB. The outcomes of this study are in harmony with research that emphasizes the role of attitude^20,23,28,29^ and PBC^4,20^, but there is no consensus on the importance of subjective norms^20^. The presumed reason for this is that the present study expanded the TPB model with latent factors, which were related to the internal motivations of the respondents. These variables were included in the model as exogenous variables, similar to subjective norms, and the results show that their effect is more significant in terms of FW. The results of this study about the effect of SN is not in harmony with international results, as a study using a systematic literature review and meta-analysis found that each of the basic TPB elements had an effect on IRFW and FWB^24^. The lack of SN effect may be explained by the fact that the impact of social media has increased significantly in recent years since the data were collected, with recent research suggesting that the trustworthiness and competence of social media celebrities have a positive effect on IRFW^30,76^. However, it is important to underline that the impact of the SN factor in the TPB model is not consistent according to international experience. One reason for this is the partially identical information content of the SN and behavior factors, and the focus on social norms when measuring the SN factor, thus ignoring the influential role of descriptive norms^99^. Descriptive norms measure the behavior of the individual’s environment, the present study measured only social norms, and therefore the results are consistent with empirical studies using the TPB framework and measuring the SN factor with similar items^100^. International empirical studies dealing with FW have also reached different results regarding the effect of SN. Examining the food wastage behavior of Indian university students, no significant effect of SN was observed^31^. A similar result was observed in the case of young Spanish consumers, by using qualitative research methods, the role of SN in influencing the intention and behavior related to FW could not be demonstrated^101^. Also, no significant effect of SN could be detected in the research conducted on a representative sample according to age and gender by using the TPB framework on the wastage of food from online food delivery^102^. HC reduces FWB, which is in line with the literature^36^. Previous studies investigating FW have already examined EC^20,26,40^, PC^4,28,35,36,46^ and PR^23,32^ factors, the present study confirmed the importance of these factors in influencing IRFW and wasteful behavior in GY and GX members. Among these factors, since the data collection of this study, the role of the way of thinking about the different environmentally-friendly behaviors has widened, which was also examined in areas such as the ease and fluency of ordering and consuming food using the respective internet platforms for food delivery. To investigate the waste of food from online food delivery, the basic TPB was expanded with food-related factors, and the increasing effect of the explained variance of the expanded construct was demonstrated^102^. The findings of this study confirmed the previously shown adverse effects of CH^4^ and UE^61^. However, the present study is the first to include and investigate the impact of EM and BO in the model extension. The findings show that EM play an important role in positively influencing IRFW. The behavior of blaming others for wasting food only plays a role in negatively influencing the intention. Analysing the habits of the generations, it was shown that GY is more likely to waste food than GX in the case of meals and leftovers, bread and other bakery products, dairy products and processed meat. There was no difference in the frequency of throwing away fresh vegetables and fruits, which is consistent with previous research^11^. Examining the moderating effect of generations in the model, the results suggest that there is no generational difference in the mechanism of action of the model factors under consideration. At the same time, the lack of generational differences is not extraordinary; examining the influence of factors influencing sustainable consumption closely related to the context of the present research, a similar behavior of the members of Generations X, Y and Z was observed^103^.

### Managerial implications

Based on the results, it is recommended that programs encouraging GY and GX members to reduce FW should aim at strengthening EC and PC, ecological thinking and PR. The results show that influence from the immediate environment and the media is not effective in itself. As Kurz et al.^16^ highlighted, there is little evidence that the consumption tastes or preferences of GY are lower than older generations’, this conclusion also applies to spending on food. GX members are autonomous individuals, occupy important positions, have more purchasing power than any other generation, are capable of making independent decisions, and their consumer behavior is essentially determined by taking care of their families and themselves^14–16^. It is recommended to organize programs dealing with the future and sustainability of the Earth and the future of children to strengthen environmental and ecological awareness in the main breadwinner groups of society, GX and GY, which can indirectly influence their IRFW and their wasteful behavior.

## Conclusions

There is a lack of research that focus on FWB of the main breadwinner groups in society, generations Y and X, this study explored the factors that influence FWB of these groups. The results show that influences from the immediate environment and the media are not effective, therefore programs dealing with the future of the Earth, children and sustainability, which strengthen environmental and ecological awareness and PR in GX and GY are recommended.

This research has some limitations. Quota sampling method does not allow calculating the sampling error, and it is risky to apply the research findings to the total community. FWB is based on respondents’ ratings, not calculated data. The data were collected over a single month. Although the questions measured food waste in general, the literature suggests that weather and seasonality of the seasons influence shoppers’ attitudes towards FW. A further research limitation is that the present study did not extend its theoretical framework to account for the effect of COVID. The analysis ignores effects between some model factors. This research did not show the effect of SN, for which several explanations were given. Future research should definitely examine the role of the SN effect in a way that takes the impact of descriptive norms in relation to FW into account. It would be worth repeating the survey in another country, with a larger sample, using random sampling method rather than quota sampling. Future research should also take food wastage linked to seasonality into account, and therefore a broader data collection is recommended. The future study could be extended to other generations to better understand generational differences. In future research, it would be worth taking the results of this study into account in such a way that the level of FW is a calculated figure. The data obtained during the measurement of the food placed in the waste bin or during the kitchen management can be included in the modeling where the calculated, real amount of FW can be included as an endogenous variable through data transformation. Separating the level of absolute waste from relative waste would help in understanding the impact of planning routines better.

## Data Availability

The datasets used and/or analyzed during the current study are available from the corresponding author upon reasonable request.
